# Supplementary material for: Ecological Network Theory Boosts Land Maxing Benefits for Biodiversity: An Example with Tropical Bee-Plant Interactions
Source: Insects. 2025 Dec 13;16(12):1269. doi: 10.3390/insects16121269 (PMC12733541; doi:10.3390/insects16121269)

Supplementary Files. **Ecological Network Theory Boosts Land Maxing Benefits for Biodiversity: An Example with Tropical Bee-Plant Interactions.** Valerie E. Peters & Elijah Cruz Cardona

Table S1. Dependent and Independent variables, sampling methods and measurement calculations.

Variable	Units	Variable Type	Measurement or Sampling Dates	Number of Visits	Observer Effort	Measurement
**Dependent**						
Shade Cover	%	numerical, continuous	5-30 Jun 2022	1	1 observer; 1 recorder	Number of Shaded Points/200
Ave DBH	cm	numerical, continuous	5-30 Jun 2022	1	1 observer; 1 recorder	(Circumference/**π**)/Total Trees
Total DBH	cm	numerical, continuous	5-30 Jun 2022	1	1 observer; 1 recorder	(**Σ**(Circumference/**π**)in 1-ha)
Ave Tree Height	m	numerical, continuous	5-30 Jun 2022	1	1 observer; 1 recorder	Individual tree height/Total Trees
Tree Species Richness	count	numerical, discrete	5-30 Jun 2022	1	1 observer; 1 recorder	Count of all tree species in 1-ha
Total Trees	individuals	numerical, discrete	5-30 Jun 2022	1	1 observer; 1 recorder	**Σ**(All individual trees in 1-ha)
Flowering Plant Species Richness	count	numerical, discrete	5 Jun-24 Jul; 7-19 Dec 2022	4	1 observer	Count of all plant species with open flowers in 1-ha
Flower abundance	count	numerical, discrete	5 Jun-24 Jul; 7-19 Dec 2022	4	1 observer	Count of all open flowers in 1-ha
Independent						
Bee species richness	count	numerical, discrete	5 Jun-24 Jul; 7-19 Dec 2022	4	3 observers X 6hr exhaustive surveys + 2 observers X 1hr X 2 honey bait stations	3 observers X 6 hrs X 4 sampling periods + 2 observers X 1hr X 2 honey bait stations X 4 sampling periods
Bee abundance	count	numerical, discrete	5 Jun-24 Jul; 7-19 Dec 2022	4	3 observers X 6hr exhaustive surveys	3 observers X 6 hrs X 4 sampling periods
Weighted connectance	NA	numerical, continuous	5 Jun-24 Jul 2022	3	3 observers X 6hr exhaustive surveys	3 observers X 6 hrs X 3 sampling periods
Modularity Q	NA	numerical, continuous	5 Jun-24 Jul 2022	3	3 observers X 6hr exhaustive surveys	3 observers X 6 hrs X 3 sampling periods
NODF (nestedness)	NA	numerical, continuous	5 Jun-24 Jul 2022	3	3 observers X 6hr exhaustive surveys	3 observers X 6 hrs X 3 sampling periods
H2' (specialization)	NA	numerical, continuous	5 Jun-24 Jul 2022	3	3 observers X 6hr exhaustive surveys	3 observers X 6 hrs X 3 sampling periods

Table S2. Raw network indices.

Farm	Type	Meliponine H2	Meliponine wC	Meliponine Q	Meliponine NODF	Entire H2	Entire wC	Entire Q	Entire NODF
FC	Pasture	0.8020407	0.09925344	0.4314169	13.72549	0.7406818	0.09006467	0.6083284	14.91165
GR	Pasture	0.4108102	0.2054753	0.3564209	28.35249	0.4363859	0.0999572	0.4952163	16.67428
AL	Pasture	0.6981549	0.1608385	0.4056122	21.2963	0.5876287	0.1111888	0.5155377	12.49589
MU	Pasture	0.6414741	0.2001825	0.1840278	11.90476	0.5628776	0.165905	0.3089665	17.88056
RE	Pasture	0.4993554	0.1441759	0.4635429	18.92677	0.5287073	0.093331543	0.5459925	15.85281
HA	Coffee	0.3942444	0.1682426	0.3717193	24.21875	0.5417657	0.1197319	0.5345751	13.01387
IA	Coffee	0.3290617	0.1704174	0.3295671	43.95714	0.3962126	0.09666944	0.3928784	15.42526
NS	Coffee	0.7538011	0.1271489	0.5920181	12.84848	0.8006755	0.1145999	0.676553	10.52974
VR	Coffee	0.5594268	0.1080376	0.559328	21.44558	0.5564905	0.08152504	0.5950972	13.57143
WL	Coffee	0.5117821	0.1682718	0.4273117	18.42105	0.4381848	0.1478609	0.3608416	21.71921

Figure S1. Bee abundance comparing coffee agroforests and silvopasture.

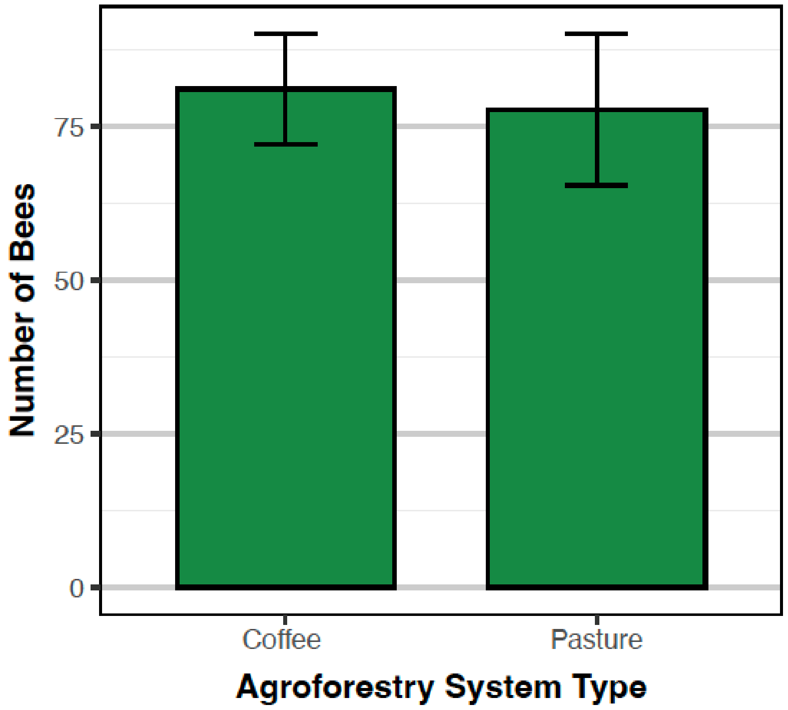

Figure S2. a. Bee abundance.

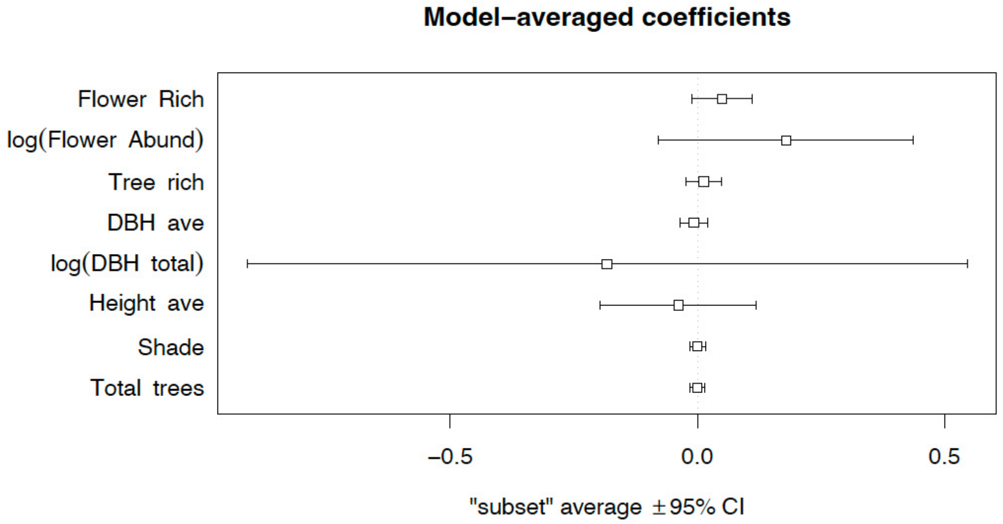

Figure S2. b. Bee species richness.

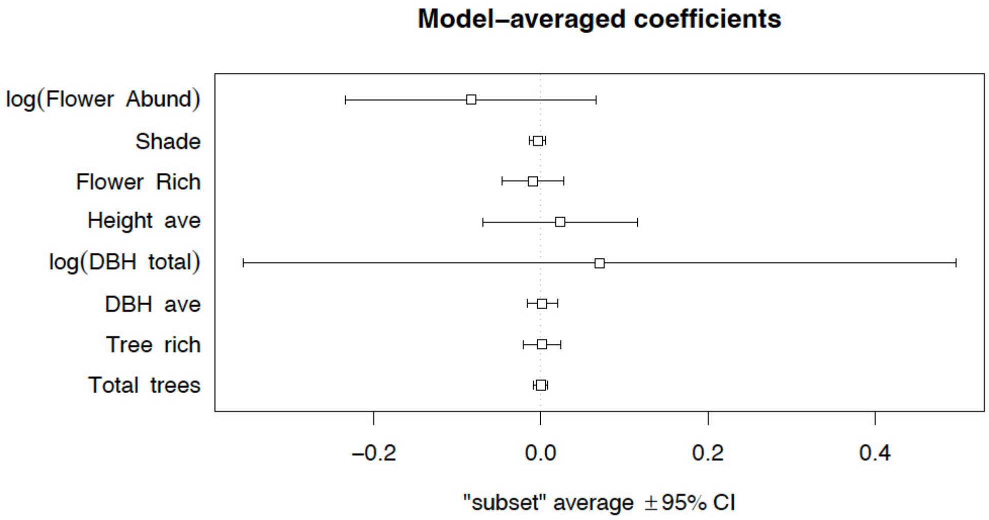

Figure S3. Species accumulation curve, comparing species richness, S, to abundance-based estimators, Chao1 and ACE.

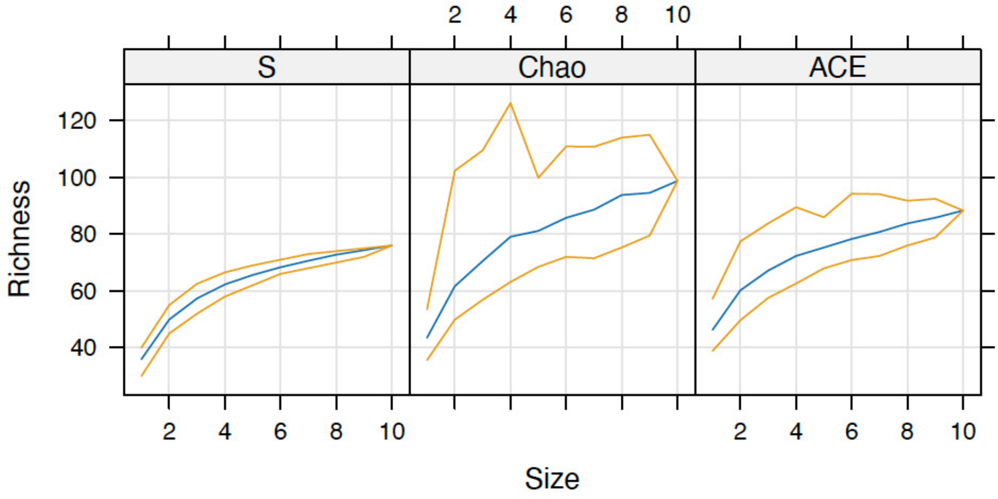

Figure S4. Distance-based redundancy ordination plot.

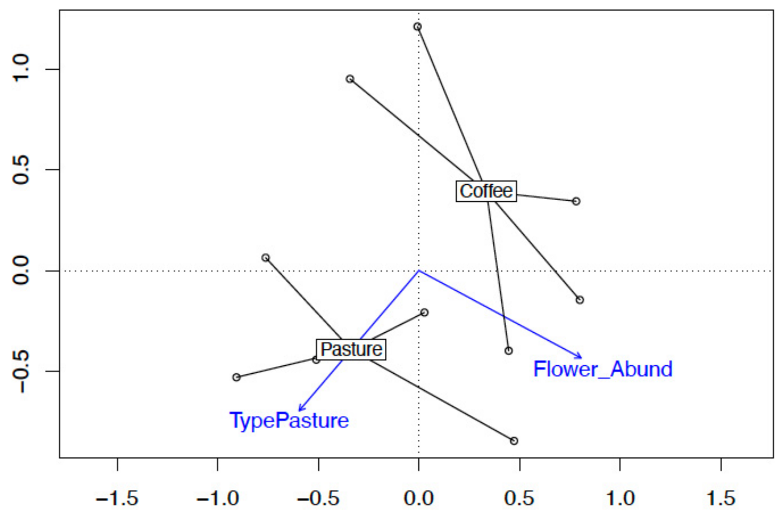

Figure S5. a. Connectance-Entire bee network.

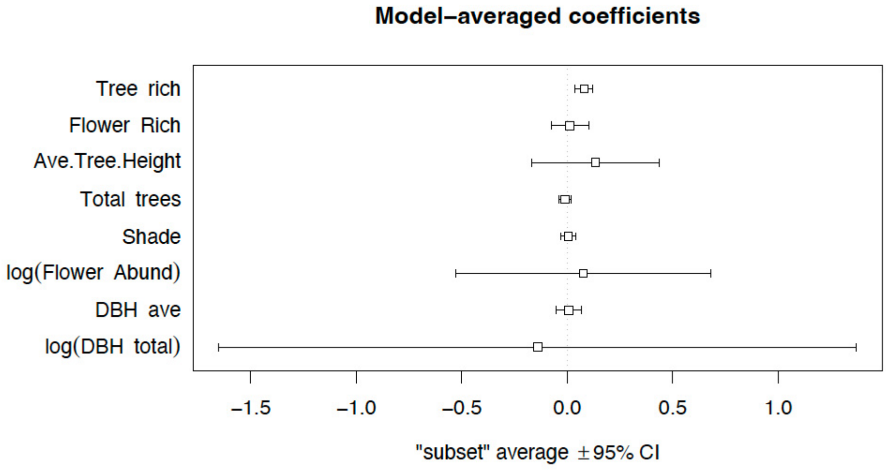

Figure S5. b. Connectance-Meliponine bee network.

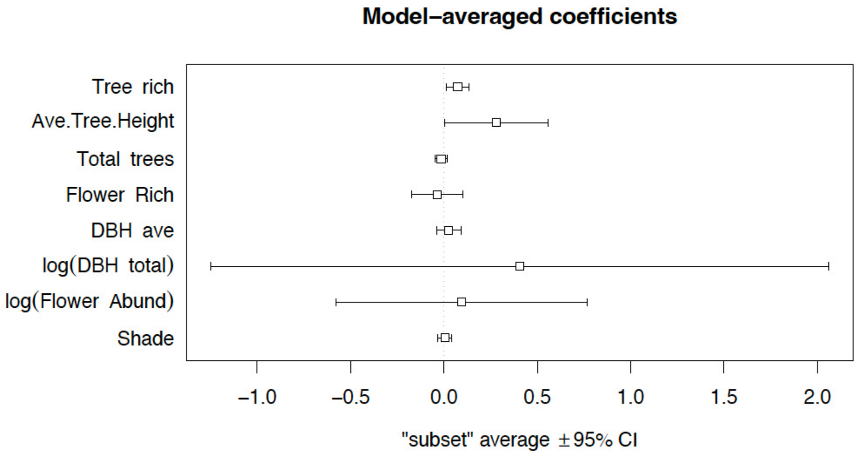

Figure S5. c. Modularity-Entire bee network.

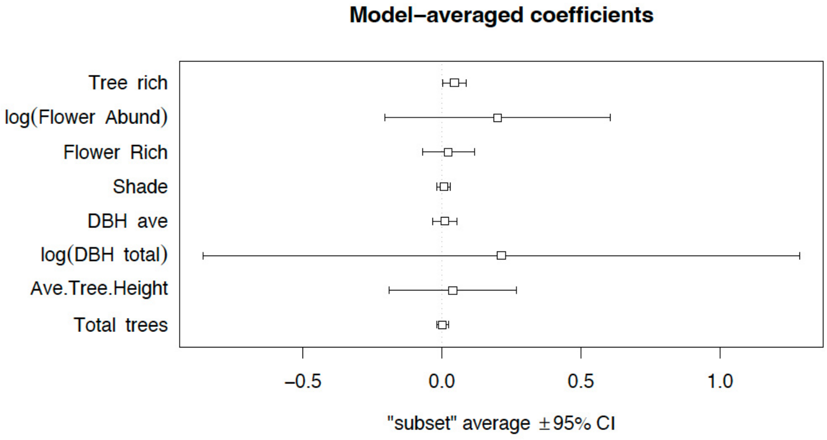

Figure S5. d. Modularity-Meliponine bee network.

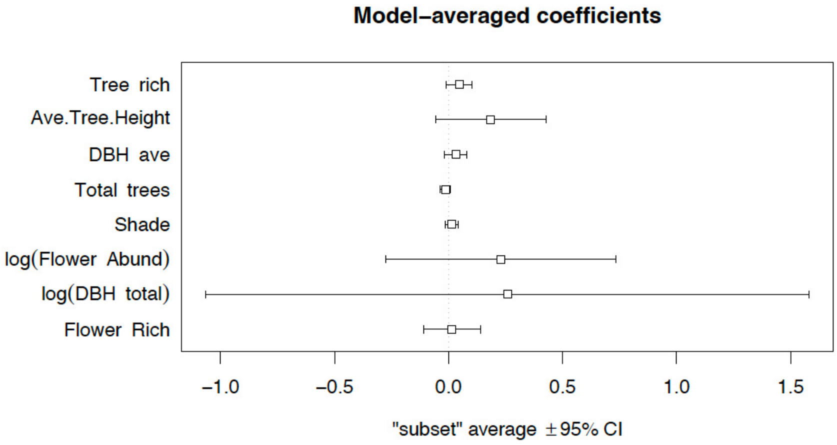

Figure S5. e. Specialization-Entire bee network.

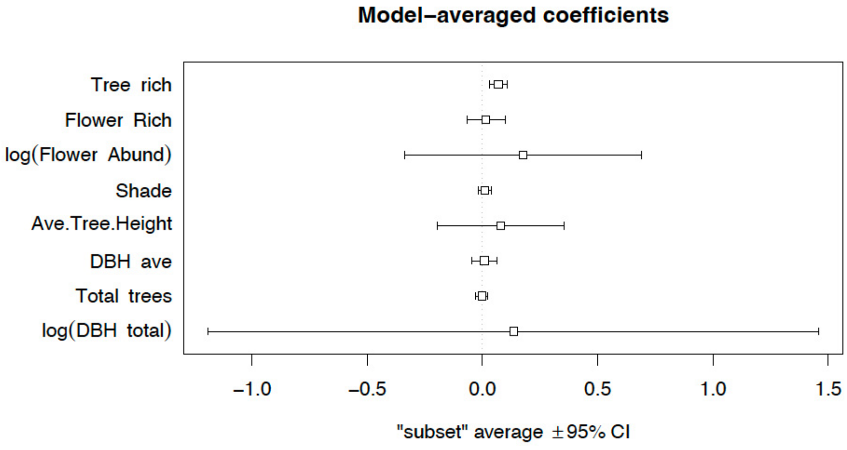

Figure S5. f. Specialization-Meliponine bee network.

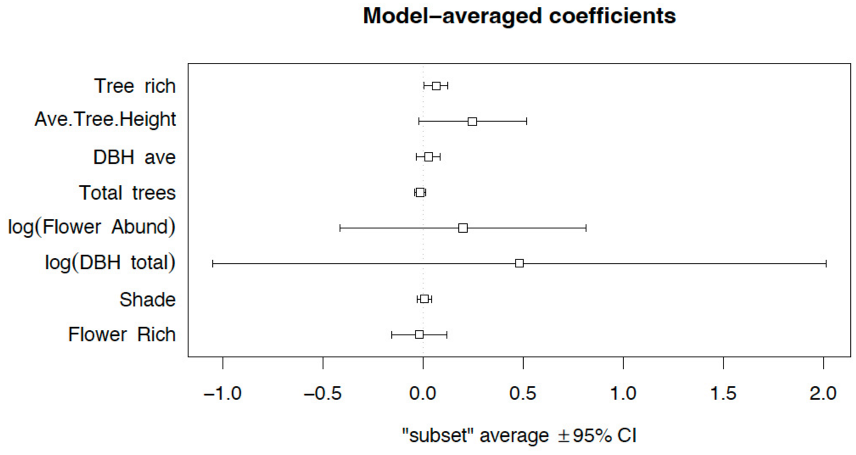

Figure S5. g. Nestedness-Entire bee network.

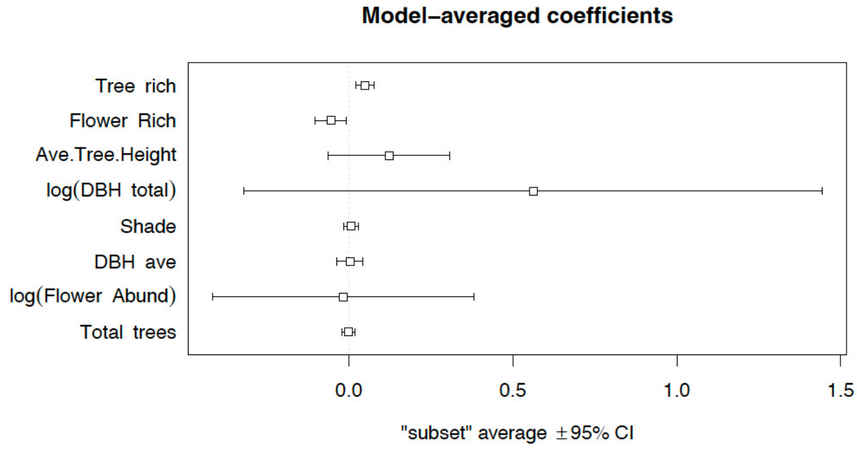

Figure S5. h. Nestedness-Meliponine bee network.

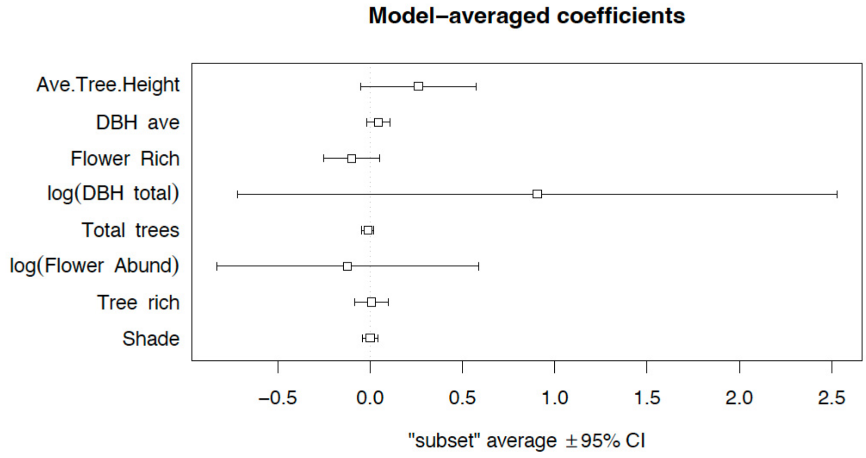

Figure S6. Entire bee and stingless bee networks quantitative modularity analysis.

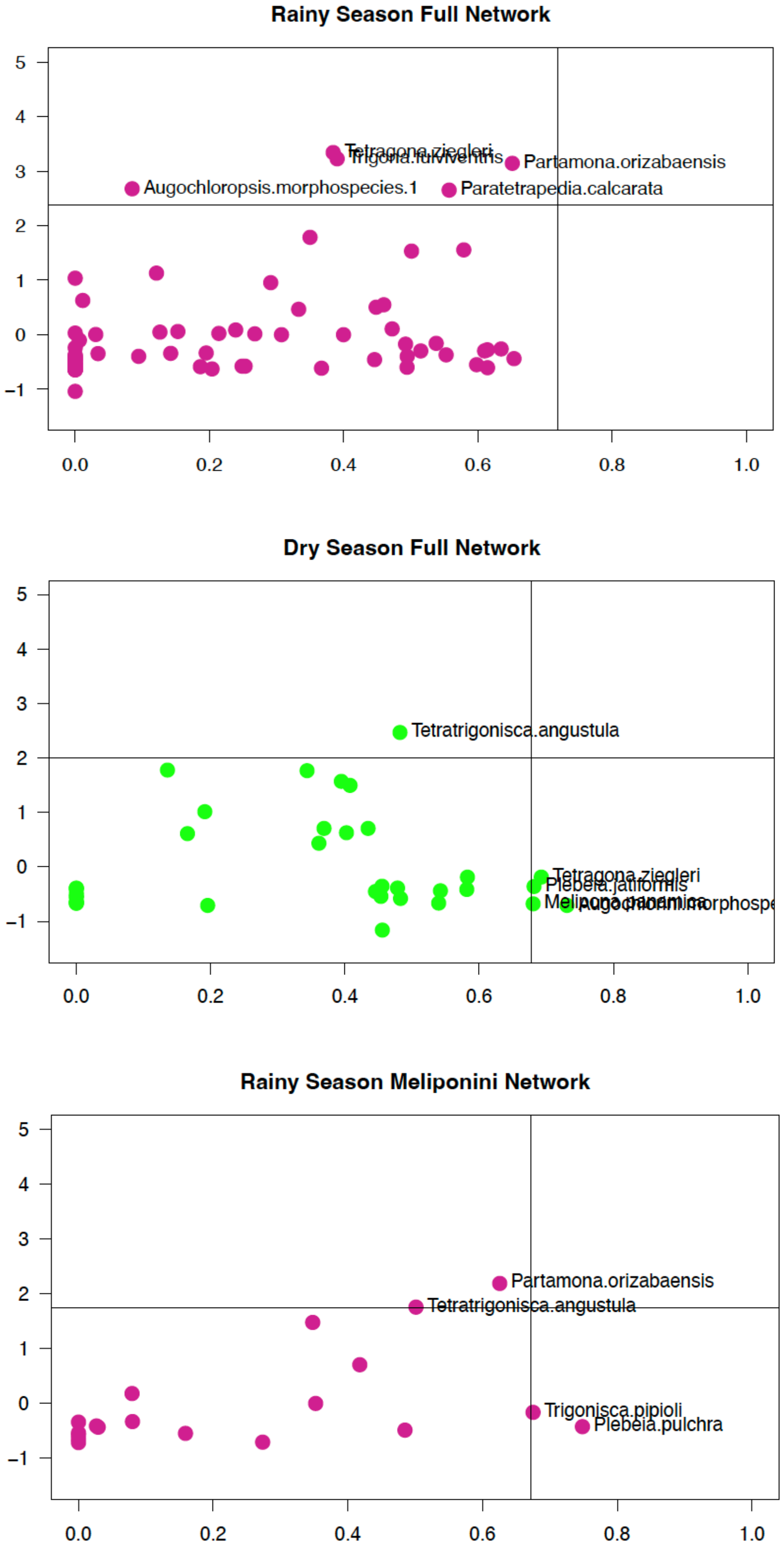

Supplement: Supplementary file 1 [file insects-16-01269-s001.zip › insects-3964001-supplementary.pdf]
